# Role of vaccine therapy in cancer: biology and practice

**DOI:** 10.3747/co.2007.158

**Published:** 2007-12

**Authors:** J. Schlom, J.L. Gulley, P.M. Arlen

**Affiliations:** * Laboratory of Tumor Immunology and Biology, Center for Cancer Research, National Cancer Institute, National Institutes of Health, Bethesda, Maryland; † Medical Oncology Branch, Center for Cancer Research, National Cancer Institute, National Institutes of Health, Bethesda, Maryland

**Keywords:** Immunotherapy, vaccine, clinical protocols, prostate cancer, cancer vaccine, combination therapy

## Abstract

Vaccines constitute a potential new therapeutic approach for a range of human cancers. Unlike other therapeutics, vaccines initiate a dynamic process in the host immune system that can be exploited with subsequent therapies. Indeed, recent preclinical and clinical studies with cancer vaccines have provided evidence that this unique therapeutic modality should lead to consideration of new paradigms in both clinical trial design and endpoints and in combination therapies. The present article reviews and sets out a rationale for these new paradigms, with a focus on prostate cancer.

## 1. INTRODUCTION

Cancer vaccines initiate a dynamic process by activating the host’s own immune system—an approach that differentiates them from other, more conventional therapies. This process could potentially influence the evaluation of patients’ responses to initial therapy and to subsequent therapies post vaccination. No therapeutic cancer vaccine has yet been approved by the U.S. Food and Drug Administration (fda), but the field of cancer vaccines is currently in a state of active preclinical and clinical investigation. Recent preclinical and clinical studies with cancer vaccines have provided evidence that this unique therapeutic modality should lead to consideration of new paradigms in both clinical trial design and endpoints and in combination therapies. Cancer vaccines may well ultimately be employed for the therapy of many types of cancer (Table I).

## 2. DISCUSSION

### 2.1 Tumour Response Versus Patient Response

In our opinion, cancer vaccines will become the therapeutic modality in which greater emphasis will be placed on “patient response” than on “tumour response,” two phenomena that are not always mutually inclusive.

Standardization of response criteria is of course critical for any given clinical trial; however, it must be emphasized that the use of only one criterion for all therapeutics, cancer types, and disease stages can be misleading. In 2000, an international committee formulated the Response Evaluation Criteria in Solid Tumors (recist) as a means of measuring tumour response 1,2. The use of recist criteria has served the oncology community well in evaluating passive therapeutic modalities such as chemotherapeutic agents and radiation therapy. With the advent of new targeted therapies, including cancer vaccines, the sole use of recist criteria has now been called into question by several cooperative groups, among others 2–5. An excellent example is found in the evaluation of sorafenib (Nexavar: Onyx Pharmaceuticals, Emeryville, CA, U.S.A.) in clinical trials of patients with advanced renal cell carcinoma. In those trials, increases in patient survival were seen with low tumour response levels and only 1 complete response from among 451 patients 6. It is clear from these results and others that recist criteria do not always adequately assess the clinical benefit to patients.

A recent article 7 characterized stable disease and longer-than-expected survival as “soft criteria” in the evaluation of cancer vaccines as compared with the “standard” or recist criteria of measuring tumour volume. In contrast, the tumour volume reduction observed in some melanoma patients treated with adoptive T-cell transfer techniques was proposed as a modality that satisfies recist criteria. The findings arising from the use of antigen-specific adoptive-transfer T-cell techniques are important and extremely innovative, and the therapy may well benefit certain subsets of patients. However, consideration must be given to the fact that, over the course of 20 years,

- no randomized trial of adoptive cell transfer techniques using either lymphokine-activated killer cells or tumour-infiltrating lymphocytes has demonstrated a statistically significant survival benefit over that seen with interleukin 2 (il-2) alone 8–10;
- no randomized trial using adoptive transfer of “antigen-specific” T cells to demonstrate a survival benefit in any patient population has been conducted; and
- studies of adoptive transfer of antigen-specific T cells have been carried out only in select institutions and have not been successfully translated to enable numerous cancer centres to carry out multicentre randomized trials 8.

In addition, other factors associated with this therapy must be considered, including toxicities and the labour- and cost-intensive efforts required by this therapy. It is thus suggested that adoptive-transfer T-cell therapy may be more applicable to patients with advanced disease, and that vaccine therapy may be more applicable to patients with a lower tumour burden or at an earlier stage in the disease process. As will be discussed shortly in this article, several randomized clinical trials have been conducted with a range of cancer vaccines that are now showing evidence of clinical benefit in terms of patient survival. It is thus important to consider different paradigms for measuring patient benefit when various therapeutics are used in varying patient populations.

### 2.2 Vaccine Trials in Prostate Cancer

An earlier review 11 listed 21 clinical trials in which a range of cancer vaccines provided evidence of clinical benefit in various patient populations. Here, we discuss recent clinical findings resulting from the use of several different vaccine types in the therapy of prostate cancer.

Prostate cancer is a disease well suited for an analysis of the efficacy of cancer vaccines for several reasons:

- Prostate tumours are relatively slow-growing.
- Recurrence is often diagnosed early in the disease state.
- The doubling time of serum prostate specific antigen (psa) acts as a surrogate marker for disease prognosis and outcome 12–14 (Arlen PM, Bianco F, Dahut WL, *et al.* Prostate-specific Antigen Working Group’s guidelines on psa doubling time. Submitted to *J Urol*).
- After definitive primary therapy (surgery or radiation, or both), few standard-of-care therapies exist that achieve long-lasting therapeutic effects.
- A range of prostate cancer–associated antigens have been identified and characterized.
- Early clinical studies have shown that patients will mount immune responses to the prostate cancer–associated antigens post vaccination.

Perhaps the most mature of the prostate cancer vaccines in terms of clinical trials is sipuleucel-T. Sipuleucel-T consists of autologous antigen-presenting cells and a fusion protein composed of prostatic acid phosphatase and granulocyte–macrophage colony–stimulating factor (gm-csf). A placebo-controlled, randomized phase iii trial using sipuleucel-T (Table II) was recently conducted 15 in patients with metastatic, asymptomatic (without cancer-related pain), hormone-refractory prostate cancer (hrpc). Progression-free survival (the primary endpoint) was not statistically significant, but overall survival between the vaccine and the placebo groups (25.9 months vs. 21.4 months) reached statistical significance (*p* = 0.01).

Another prostate cancer vaccine employed in advanced clinical trial testing is Gvax (Cell Genesys, San Francisco, CA, U.S.A.). Gvax consists of two irradiated allogeneic prostate cancer cell lines engineered to secrete gm-csf. Its mode of action is proposed as the uptake of the *ex vivo* X-irradiated tumour cells by apc and cross-presentation of tumour-associated antigens to T cells in draining lymph nodes. Two phase ii clinical studies have now been completed in patients with asymptomatic metastatic hrpc 16–18. In the first trial (*n* = 34), Gvax was given at two dose levels. In the second trial, using the same patient population (*n* = 80), vaccine was given to five dose-level cohorts: two low-dose cohorts (*n* = 33), one intermediate-dose cohort (*n* = 25), and two high-dose cohorts (*n* = 22). In the low-dose cohorts, the median survival of patients was 23.1 months; in the intermediate-dose cohort, it was 20.0 months. The median survival of the high-dose cohorts is 35 months (Table II). No dose-limiting toxicities were observed in either trial.

Onyvax-P vaccine (Onyvax, London, U.K.) is another whole tumour-cell vaccine for prostate cancer that is also demonstrating promising clinical results. Onyvax-P consists of three irradiated allogeneic prostate cell lines. These cell lines have been shown to express a broad range of prostate- and prostate cancer–associated antigens, in a manner similar to the Gvax vaccine. A phase ii Onyvax-P trial has now been completed in hrpc patients 23. Of 26 patients, 11 showed a statistically significant prolonged reduction in psa velocity, and no patient had a statistically significant increase in psa velocity post vaccination. Mean time to tumour progression was 58 weeks as compared with the approximately 28 weeks seen with recent studies of other agents and with historical control values. Artificial neural network analysis of the immunologic profiles of cytokines correlated with the psa velocity responses 23.

Several trials with vaccines using poxvirus vectors have exhibited evidence of clinical benefit. Poxviruses [*Vaccinia* (rV–), modified *Vaccinia* strain Ankara (mva), and fowlpox (rF–)] have the ability to accept and express multiple transgenes and can thus be engineered to express not only tumour-associated antigens, but also various immunostimulatory molecules. Modified *Vaccinia* Ankara is a replication-incompetent *Vaccinia* virus 24. The recombinant mva called TG4010 expresses both Muc1 (a higher-molecular-weight mucin that is overexpressed in most carcinomas, including prostate carcinoma) and il-2. A randomized phase ii study 24 of TG4010 has been completed in prostate cancer patients with biochemical progression and no evidence of metastatic disease after local therapy (psa doubling time of less than 10 months). Of 38 patients, 27 (71%) had a lengthened psa doubling time after vaccination.

A U.S. National Cancer Institute (nci)–sponsored Eastern Cooperative Oncology Group randomized phase ii trial was then carried out using two different psa poxvirus vectors in various prime-and-boost regimens: recombinant fowlpox rF–psa (F) alone or combined with rV–psa (V) in patients (*n* = 64) with biochemical progression after local therapy for prostate cancer. At the 2-year follow-up, median time to psa or clinical progression, or both, was 9.2 months in the cohort that received four vaccinations with F (FFFF), 9.0 months in the cohort that received a V vaccination after three F vaccinations (FFFV), and not yet reached in the cohort that received three F vaccinations after a V vaccination (VFFF) 25. Moreover, a recent update report 26 on the trial, with a median follow-up of 50 months, revealed a median time to psa progression of 9.2 months and 9.1 months for the FFFF and FFFV cohorts respectively, and 18.2 months for the VFFF cohort.

The U.S. nci has now developed recombinant *Vaccinia* and fowlpox vectors containing the transgenes for psa and three human co-stimulatory molecules (CD80, intracellular adhesion molecule 1, and lymphocyte function–associated antigen 3, designated “ tricom”) 27,28. Recent phase i and ii trials involving patients with metastatic and locally advanced prostate cancer have shown clinical responses and drops in serum psa 27,28. A multicentre randomized phase ii study 19 in 125 patients with metastatic androgen-independent asymptomatic prostate cancer did not meet its primary endpoint of progression-free survival, but the patients’ overall survival data are currently being accumulated, with provocative results (Table II). Median overall survival thus far is 16.3 months for the control cohort (fowlpox wild-type vector, *n* = 41) as compared with 24.4 months for patients receiving psa–tricom vaccines (*n* = 84).

Vaccine trials in prostate cancer illustrate the progress being made in clinical vaccine therapy. However, they represent only one example of the strides being made in treating a range of human carcinomas. Ongoing progress in pancreatic cancer 29,30, lymphoma 31, melanoma 32,33, lung cancer 34, and other tumour types are also providing evidence of vaccine efficacy in clinical trials 11.

### 2.3 Separating Vaccine Efficacy from Poor Clinical Trial Design

Clinical studies have now validated what had previously been predicted by many:

- A direct correlation is evident between the cancer patient’s ability to mount an immune response to a vaccine and the length of time since the patient’s last therapy, and
- an inverse correlation is evident between the cancer patient’s ability to mount an immune response to a vaccine and the patient’s number of prior therapies 35,36.

A classic example of the distinction between a vaccine’s potential efficacy and a poor clinical trial design is evidenced by an ill-conceived corporate trial in which tricom-based vaccines [carcinoembryonic antigen (cea)–Muc1–tricom, called Panvac-VF (Therion Biologics, Cambridge, MA, U.S.A.)] were administered to patients with metastatic pancreatic cancer who had already failed prior gemcitabine therapy 37. As many predicted, this trial failed to meet its primary endpoint of overall survival. Poor clinical trial design was clearly illustrated by

- the median overall survival of less than 3 months in this patient population;
- the fact that only one drug combination has been approved by the U.S. fda for the therapy of pancreatic cancer (gemcitabine plus erlotinib), which extended survival by 0.4 months; and
- twelve randomized trials of various fda-approved drug combinations (including bevacizumab plus gemcitabine) have failed to extend survival in this patient population.

Thus, failure of a phase iii vaccine trial in this patient population should foremost be considered a failure in company-sponsored clinical trial design.

In contrast, a U.S. nci-sponsored trial of a cea–tricom-based vaccine has recently been completed 38. Patients (*n* = 58) with progressing cea*-*expressing cancers (predominantly colorectal and lung) were accrued into several cohorts. They received rV– and rF–cea–tricom vaccines with or without gm-csf. Among these patients, 40% had stable disease for at least 4 months, and 14 had prolonged stable disease (more than 6 months). In 11 patients, serum markers declined or stabilized, and 1 patient had a pathologic complete response. A clear trend was also observed in overall survival in cohorts receiving gm-csf with vaccine and in patients who had higher frequencies of cea-specific T-cell responses 38.

This protocol also revealed a novel situation: Patients who achieved stable disease after 6 monthly tricom vaccinations went on to receive vaccines every 3 months. Of those patients, 12 then progressed and were changed back to monthly vaccinations. Subsequently, 6 of the 12 again reverted to disease stabilization 38. This phenomenon of first stabilizing on a therapeutic, and then progressing, and then re-stabilizing when dose scheduling is changed, underscores the paradigms that must be investigated in the use of this relatively new therapeutic modality.

### 2.4 New Paradigms for Combination Therapies

The five strategies of combination therapies, which are described in this subsection and which are being employed with cancer vaccines, have been validated in preclinical models, and several have provided preliminary evidence of clinical benefit (Table III). As the field matures, and if and when the U.S. fda approves one or more vaccines or other immune stimulants, progress in this area will undoubtedly be accelerated.

#### 2.4.1 Conventional Combination Therapy

In most combination therapies that use two or more chemotherapeutic agents or a chemotherapeutic agent and a targeted therapy, each agent works individually with the goal of additive antitumour effects. Additive effects have been demonstrated in numerous preclinical models combining vaccines with chemotherapeutic agents. Preclinical and early clinical studies alike have highlighted the following important phenomenon: although vaccines are less effective in patients heavily pretreated with chemotherapy before vaccine use, no detrimental effects in the immune response to vaccine have been seen in patients when vaccine is given in combination with chemotherapeutic agents such as 5-fluorouracil 39 and docetaxel (even when given with a steroid) 40.

#### 2.4.2 Vaccine in Combination with Agents That Affect the Host Immune System

Many reagents that act either as immune stimulants and adjuvants or as inhibitors of immunoregulatory cells or molecules are currently being used in combination with vaccines. These reagents have been shown in multiple preclinical models to enhance the efficacy of vaccine. Cytokines are well-proven to enhance the immune response. For example, gm-csf has now been demonstrated in numerous clinical trials to enhance vaccine efficacy 15,18,29,30,38. Other immune stimulants such as Bacillus Calmette–Guerin, CpG motifs, and imiquimod are also currently being employed clinically with vaccines. One agent that is showing great promise is the monoclonal antibody anti–cytotoxic -4) T lymphocyte–associated antigen 4 (anti-ctla 41,42—ctla-4 being the molecule expressed by antigen-primed activated T cells. Although the exact mechanism by which this agent works with vaccine has never been shown clinically, preclinical studies have clearly shown that the use of anti-ctla-4 with vaccines results in higher-avidity antigen-specific T cells 43.

Based on numerous preclinical studies and recent clinical studies, it has become apparent that control of immune inhibitory entities will play an important role in vaccine-mediated therapies. Preclinical and clinical studies 44–46 have shown that denileukin diftitox (a fusion protein of diphtheria toxin and il-2) can kill CD4/CD25/Foxp3 regulatory T cells (“T regs”) and enhance vaccine efficacy in inducing greater T-cell responses. The chemotherapeutic agent cyclophosphamide has also been shown in both preclinical and clinical studies to enhance vaccine efficacy. Cyclophosphamide reduces not only the number of T regs, but also their functionality 47,48.

#### 2.4.3 Multiple Vaccine Therapies

The use of multiple vaccines may ultimately prove advantageous because

- various types of vaccines have been shown to enhance various entities of the host immune system—that is, to produce greater increases in CD4, CD8, or natural killer cells, or antibodies.
- each vaccine can carry different tumour-associated antigens.
- vaccine therapy has been associated with limited toxicities.
- some vaccines will induce host immune responses to the vaccine vehicle (viral vectors, for example), limiting their continued use.

#### 2.4.4 Dose Scheduling of Vaccine with Other Therapies

The administration of a vaccine initiates a dynamic process of host immune response that may be exploited in subsequent therapies. This is perhaps the most unique feature of vaccine therapy.

Among the clinical studies that have provided evidence of this phenomenon are three randomized clinical trials in prostate cancer. The U.S. nci has now completed studies with a diversified prime–boost strategy involving priming with psa-*Vaccinia* recombinants (rV–psa plus rV–CD80) followed by multiple booster vaccinations with psa-fowlpox recombinants. In the first trial 40, 28 patients with metastatic androgen-independent prostate cancer were randomized to receive vaccine alone or vaccine plus docetaxel. Patients on the vaccine arm alone were allowed to cross over to receive docetaxel at the time of progression. Median progression-free survival on docetaxel was 6.1 months for the crossover patients as compared with 3.7 months in the patients that started on docetaxel with the same regimen and the same patient population at the same institution. Similar findings were observed in a separate trial of the sipuleucel-T vaccine. In the randomized multicentre sipuleucel-T study described earlier 12, patients in both the vaccine arm (*n* = 51) and the placebo arm (*n* = 31) went on to receive docetaxel at progression. A striking and statistically significant (*p* = 0.023) increase in overall survival was observed with docetaxel treatment in patients who received the vaccine as compared with those who received placebo (Table II) 20.

In another phase ii trial at the U.S. nci, 42 patients with nonmetastatic hrpc and rising levels of psa were randomized to receive either vaccine (rV–psa/CD80 prime and rF–psa boosts; *n* = 21) or nilutamide (*n* = 21), an androgen receptor antagonist. After 6 months, patients with a rising psa and no evidence of metastasis by radiographic criteria were allowed to “cross over” and receive a combination of both therapies 21. From the initial randomized population (*n* = 21 per cohort), 5-year overall survival was 38% for the patients who received nilutamide first (nilutamide alone or nilutamide and then vaccine) as compared with 59% for the patients who received vaccine first (vaccine alone or nilutamide plus vaccine; Table II .) 22

The foregoing trials and others 49,50 at different institutions using different vaccines and patient populations have all provided evidence of the same phenomenon: patients who receive vaccine (and who mount immune responses as a result) have enhanced outcomes with subsequent therapies. This phenomenon may be attributable to one or a combination of factors:

- The subsequent therapy may be reducing suppressor cell populations, thus allowing for enhancement of earlier-established T-cell responses.
- The subsequent therapies may be lysing some tumour cells that are then, as a consequence of cross-priming, activating relatively dormant T cells to elicit an antitumour response.
- The subsequent therapy may alter the phenotype of tumour cells or, as in androgen deprivation therapy, enhance T-cell levels (next subsection).

#### 2.4.5 Phenotype Alterations in Tumour Cells

Yet another paradigm to exploit in vaccine combination therapies is the phenomenon of certain standard-of-care therapeutics actually altering the phenotype of tumour cells to render them more susceptible to lysis mediated by T cells. Phenotype alteration has been shown in a series of preclinical studies involving murine models *in vivo,* and in a large series of human tumour cell lines *in vitro* 51–53. Sublethal doses of X-irradiation delivered to tumours have been shown to upregulate one or a combination of tumour-associated antigens, *fas*, or adhesion molecules, or to downregulate anti-apoptotic genes (or both), subsequently rendering the phenotypically altered tumour cells more susceptible to antigen-specific T cell–mediated lysis. Similar alterations of tumour-cell phenotype and subsequent susceptibility to T cell–mediated lysis have also been induced by sublethal doses of certain chemotherapeutic agents 53. These findings may ultimately lead to another paradigm shift in vaccine combination therapies: That is, when a drug or radiation treatment fails to produce a response because of inability to lyse tumour cells, such a treatment may continue to be used with vaccine therapy because of its ability to augment vaccine-induced T-cell lysis of tumour.

## 3. CONCLUSIONS

Here, we have reviewed only a few of the vaccine vehicles that are currently being used with evidence of clinical benefit. Allogeneic whole tumour cells, peptide- or protein-pulsed antigen-presenting cells (including dendritic cells), recombinant dna and viral vectors, and recombinant *Saccharomyces* (yeast) are all in active clinical trial development. Moreover, a vast array of newly defined potential tumour-associated targets are available for cancer vaccine development, including those involved in the neoplastic or tumour progression processes.

In our view, the field of therapeutic cancer vaccines is alive and thriving. As new paradigms in clinical trial design and endpoints and in combination therapies are realized, cancer vaccines may ultimately become a standard component in the management of several types of cancer.

## Figures and Tables

**TABLE I:** New paradigms for vaccine efficacy as compared with those for conventional therapeutics

Efficacy of vaccines can be enhanced by biologic adjuvants
Tumour cell phenotype can be altered to enhance vaccine efficacy
Vaccines induce a dynamic process (important in combination scheduling)
As compared with tumour response (recist), patient response(survival) may be the more important criterion for efficacy

recist = Response Evaluation Criteria in Solid Tumors.

**TABLE II:** Evidence of vaccine-mediated enhanced survival in prostate cancer clinical trials

Reference	Patient population	Study arm	Patients (*n*)	Result
Small *et al.*, 2006^15^	Metastatic asymptomatic hrpc	apc/pap/gm-csf Placebo	82 45	Median OS: 25.9 mo. 21.4 mo.
Simons *et al.*, 2006 16; Small *et al.*, 2006 17; and Simons *et al.*, 2005 18	Metastatic asymptomatic hrpc	Whole tumour cell (Gvax): high dose mid-/low dose	22 25/33	35 mo. 20.0/23.1 mo.
Kantoff *et al.*, 2006 19	Metastatic hrpc	psa/tricom Vector control	84 41	24.4 mo. 16.3 mo.
Petrylak *et al.*, 2006 20	Metastatic hrpc	Vaccine (apc/pap/gm-csf), then docetaxel Control, then docetaxel	51 31	OS: 50% (approx.) at 36 mo. 25% (approx.) at 36 mo.
Arlen *et al.*, 2005 21; and Madan *et al.*, 2007 22	Non-metastatic hrpc	Vaccine (rv-psa/B7.1, rf-psa), with or without nilutamide a Nilutamide, with or without vaccine	21 21	59% at 5 years 38% at 5 years

a Nilutamide is a second-line hormone receptor antagonist.

hrpc = hormone refractory prostate cancer; apc = antigen-presenting cell; pap = prostatic acid phosphatase; gm-csf = granulocyte–macrophage colony stimulating factor; os = overall survival; psa = prostate-specific antigen.

**TABLE III:** Cancer vaccine combination therapies

Conventional combination therapy Each agent has independent antitumour effects Vaccine plus agent or agents that augment the host immune response Immune potentiators: Cytokines—for example, granulocyte–macrophage colony stimulating factor, interleukin 7, interleukin 15 Danger signals—for example, CpG motifs, Bacillus Calmette–Guerin, imiquimod cream Androgen deprivation therapy Regulation of immune inhibitors: Denileukin diftitox Cyclophosphamide Anti-ctla-4 antibody Multiple vaccine therapies Diversified prime–boost regimens—for example, DNA–modified *Vaccinia ankara, Vaccinia–*fowlpox Combinations targeting various tumour antigens Phenotypic alteration of tumour cells to enhance T cell– mediated lysis Irradiation of tumour or tumour cells: External-beam Radiolabelled MAb Chelated radionuclide Certain chemotherapeutics: 5-Fluorouracil, cisplatin Dose scheduling of vaccine in relation to combination therapy Vaccines initiate a dynamic process, which can be potentiated by subsequent therapies Several reports suggest better clinical efficacy of certain agents post vaccine: Docetaxel Androgen receptor antagonist (nilutamide) Other chemotherapeutics
